# COVID19 infection in a patient undergoing treatment for Paroxysmal Nocturnal Hemoglobinuria (PNH) with Ravulizumab

**DOI:** 10.1186/s12959-021-00330-6

**Published:** 2021-10-21

**Authors:** Sufana Shikdar, Azra Borogovac, Elabdallah Mohamad, Mohamad Khawandanah

**Affiliations:** 1 Department of Hematology/Oncology, Stephenson Cancer Center, University of Oklahoma Health Sciences Center, 73104 Oklahoma City, United States OK; 2 Department of Internal Medicine, University of Oklahoma Medical Center (OUMC), 73104 Oklahoma City, United States OK; 3 Hematology/Oncology fellow, Department of Medicine, Division of Hematology-Oncology Stephenson Cancer Center, University of Oklahoma Health Sciences Center, 800 NE 10th St., 6th Floor, OK 73104 Oklahoma City, United States

**Keywords:** COVID19, Paroxysmal nocturnal hemoglobinuria, Complement inhibitor

## Abstract

**Background:**

In the recent COVID19 pandemic, patients with hematological disorders were considered at high risk for severe disease. Limited data is available regarding the course of COVID19 infection in this subgroup.

**Case Presentation:**

We describe a case of a 32-year-old man with paroxysmal nocturnal hemoglobinuria (PNH) undergoing treatment with ravulizumab (Ultomiris) who presented with COVID19 infection. He experienced only mild symptoms and had a rapid recovery from COVID19 infection.

**Conclusion:**

This case may demonstrate the beneficial effects of ravulizumab on complement mediated inflammatory damage linked with COVID19 infection especially in PNH patients.

## Background

Coronavirus disease 2019 (COVID-19) has become a global pandemic with at least 229.3 million confirmed cases and a total of 4,700,000 deaths worldwide as of September 2021 [1]. In December 2019, an outbreak of COVID-19 disease was detected in China caused by the Severe Acute Respiratory Syndrome Coronavirus-2 (SARS-CoV-2). The clinical manifestation of COVID-19 is characterized by respiratory distress, and in more severe cases can progress towards acute respiratory distress syndrome (ARDS) and death [2]. COVID-19 infection carries a potentially fatal risk, especially with an immunocompromised state and multiple comorbidities [3]. Several mutations of SARS-CoV-2 have been reported so far, and most recently a new mutation in the Delta variant (B.1.617.2 lineage) was found which seems to be highly contagious and is now the dominant variant globally [4]. Meanwhile, on December 11, 2020, FDA approval of the Pfizer-BioNTech in individuals 16 years of age and older vaccine has opened a new door to fight against the global pandemic; currently, we have three FDA approved vaccines available in the US [4]. Even though the COVID vaccine is highly effective at preventing severe illness, they do not give full protection in preventing and transmitting the COVID-19 infection.

COVID-19 infection causes hyperactivation of the complement system and excessive inflammatory response leading to worsening lung injury and poor clinical outcomes [2, 5]. The interaction between the complement system and COVID-19 infection raises the possibility that immunosuppression could be a promising approach to inhibit the consequences of complement mediated inflammatory destruction in COVID-19 infection. Moreover, patients with PNH may be vulnerable to COVID-19 complications due to impaired immune status [6]. Based on this immunological rationale, it was speculated that the therapeutic use of complement inhibitors might be an effective strategy to control systemic inflammation in COVID-19 infection.

Although complement blockade strategies are being prospectively studied in clinical trials and reported in case reports, no cases of PNH with COVID-19 infection managed with complement blocker agents have been reported [7, 8]. Herein, we report the first case of COVID-19 pneumonia in a patient with PNH on treatment with intravenous ravulizumab, a complement component C5 inhibitor. He had a favorable clinical course linked to COVID19 infection and was discharged without any complications.

## Case presentation

Our patient was a 32-year-old Nigerian immigrant male who presented around September 2018 with a clinical picture of severe aplastic anemia. The patient received one cycle of Horse antithymocyte globulin (ATG; 40 mg/kg once daily for 4 days); two weeks of prednisone (100 mg orally twice a day, then tapered), and six months of eltrombopag (150 mg orally daily) and cyclosporin A (10 mg/kg orally daily). The diagnosis of combined PNH and aplastic anemia due to bone marrow failure, hemolysis, and detection of PNH clone in addition to aplastic hypocellular marrow was made, and the patient was started with eculizumab for 5 cycles, then switched to ravulizumab around March 2019 with the last dose in February 2020. He remained profoundly pancytopenic and he was waiting for an allogeneic transplant.

**Figure 1:**
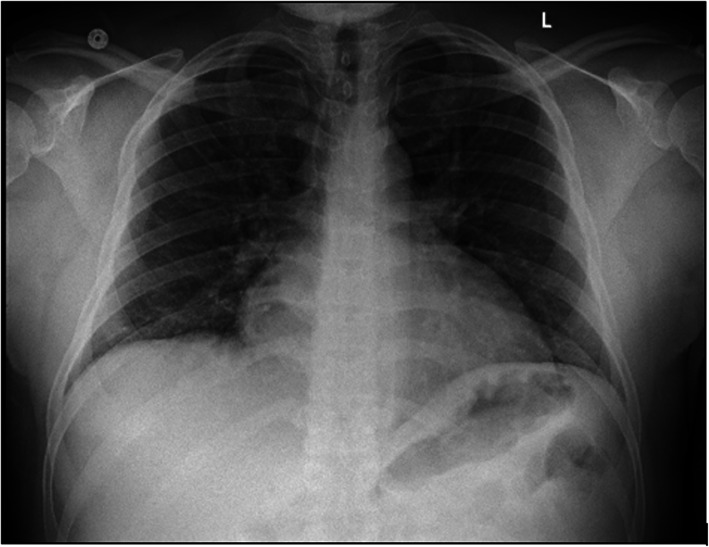
**Fig. 1** Chest Xray on admission: No infiltrates noted with COVID19 infection

He presented in March 2020 with fever, runny nose, dry cough, and altered taste (dysgeusia) for 4 days. Physical examination revealed normal blood pressure, fever (38.3 °C), tachypnea (30 breaths per min) with baseline oxygen saturation of 95 % in room air. The COVID19 test using targeted rich multiplex polymerase chain reaction of a nasopharyngeal swab came back positive for SARS-CoV-2 infection. A chest radiography did not show infiltrates (Fig. 1). Infectious workup including blood and urine cultures were negative. Respiratory viral panel was negative for influenza A and B. Laboratory tests revealed WBC 1.93 cells/mm3, absolute neutrophil count (ANC) 950 cells/mm3, Hb 8 gm/l, Platelet 37,000/mm3; increased levels of acute phase reactants, including CRP (108 mg/l; normal range <5 mg/l), ferritin (3355 ng/ml; normal range 10–322 ng/ml), serum lactate dehydrogenase (364 U/l; normal range 112–236 U/l), and fibrinogen (615 mg/dl; normal value 150-450 mg/dl). The patient was treated with azithromycin, hydroxychloroquine, and prophylactic enoxaparin. He required no oxygen, remained afebrile, and oxygen saturation was maintained around 95 % in room air. He demonstrated a rapid and progressive improvement in his symptoms and was discharged from the hospital 4 days after diagnosis with no immediate complications and no evidence of breakthrough hemolysis.

## Discussion and conclusions

PNH is a clonal disorder of hematopoietic progenitor cells caused by an acquired mutation of the X-linked phosphatidylinositol glycans class A (PIG-A) gene [9]. The absence of glycosylphosphatidylinositol (GPI) anchored complement regulatory proteins CD55 and CD59 from the membrane of circulatory cells is responsible for the activation of the complement system on the surface of the red cell membrane. This leads to complement mediated intravascular hemolysis, activation of platelets, and the coagulation cascade resulting in a hypercoagulable state [9]. PNH, although rare, can be fatal and includes an increased risk of thromboembolism and severe end-organ damage. Approximately, 35 % of patients die within five years if untreated due to thrombosis and related complications [10].

There is a close relationship between PNH and aplastic anemia (AA) especially when PNH evolves into bone marrow hypoplasia, which is a hallmark of AA. An overlap entity can be the presenting symptom i.e., dual diagnosis. Three presentations lead to overlap or dual diagnosis: (1) aplastic anemia on original presentation then the discovery of PNH clone, (2) PNH presentation then evolution to aplastic anemia, and (3) Aplastic anemia and PNH dual diagnosis on presentation [11]. Our patient presented with the dual diagnosis of PNH manifested by hemolysis and jaundice in addition to profoundly aplastic bone marrow.

Ravulizumab (ALXN1210; Alexion Pharmaceuticals, Inc) is a second generation humanized monoclonal antibody that prevents complement protein 5 (C5) cleavage and activation, ultimately blocking membrane attack complex (MAC) formation in the complement pathway [12]. Ravulizumab is now approved by the FDA to treat PNH. It has an extended 8-week maintenance dosing interval and reduces the need for frequent drug administration with subsequent improvement in symptom control and better quality of life.

The complement system activation is a critical component in the sequelae of COVID19 infection. Evidence suggests that severe outcomes in COVID19 infection are attributed to the excessive activation of the complement cascade leading to acute lung injury and associated with an increased prothrombotic state [2, 5, 13, 14]. Notably, C5a concentration was noted to be higher in patients with COVID19 infection [2, 14]. Based on these observations, complement component blockers could be used as potential therapeutic targets in COVID-19 patients.

Several clinical trials have been ongoing to target complement mediated inflammatory response in the emerging COVID19 outbreak. An anti C3 agent, compstatin analog Cp40/AMY-101 has shown efficacy in complement mediated severe ARDS in COVID19 patients, and a phase II clinical trial is ongoing [7]. In addition, a multicenter phase II/III trial using a monoclonal neutralizing anti-C5a antibody (IFX-1) is recruiting patients with severe COVID19 pneumonia [8]. Preliminary PK/PD analysis of ALXN1210-COV-305 phase 3 trial found that patients with severe COVID-19 infection treated with modified ravulizumab dosing regimen resulted in improved clinical outcomes [15, 16]. Alexion also initiated a clinical trial in critical COVID19 patients on a related drug, eculizumab (NCT04288713) [17]. A proof-of-concept study by Annane et al. found survival benefits with eculizumab in patients with severe COVID-19 [18]. Clinical and experimental research is ongoing to optimize therapeutic interventions targeting the complement system [19–22]. For example, zilucoplan, an anti-C5 drug, is being evaluated in a phase 2 ACCORD trial for hospitalized COVID-19 patients [19]. Also, the TACTIC-R trial is currently assessing the efficacy of ravulizumab among Pre-ICU COVID-19 patients [20]. In addition, Multi-center Pilot Trial (PROTECT-COVID-19) is evaluating the efficacy of conestat alpha, the C1 esterase inhibitor in severe COVID-19 infection [21], and I-SPY COVID-19 trial is investigating the benefit of anti-MASP-2 antibody Narsoplimab in critically ill COVID-19 patients [22]. While these trials posit a new potential treatment strategy, the therapeutic use of complement inhibitors in COVID-19 immunocompromised patients is limited. Few case reports have shown a promising effect of complement inhibitors in COVID-19 patients. Eculizumab used in the patients with thrombotic microangiopathic anemia in the setting of COVID19 showed dramatic improvement in kidney function [23]. Another patient with a transplanted kidney who was on Eculizumab for the atypical hemolytic uremic syndrome (HUS), was treated for COVID19 infection and had a full recovery [24]. Similarly, a patient with PNH diagnosed with COVID-19 pneumonia showed fast recovery with the initiation of eculizumab [25].

Immunocompromised patients are at higher risk for severe COVID19 infections and may experience prolonged hospitalization due to COVID19-related morbidity, and mortality. Additionally, administration of most scheduled anti-cancer treatments may be delayed or interrupted. In our case, the continuation of ravulizumab during COVID19 infection may have assisted a prompt recovery by attenuating complement activation.

The CDC recommends adopting strategies to minimize the risk of COVID19 exposure, such as limiting contacts between patients and healthcare providers. Ravulizumab has a longer therapeutic window and therefore a less frequent dosing schedule than eculizumab. We recommend against interrupting or holding the dose of ravulizumab even during systemic inflammation to combat complement mediated tissue damage and multiorgan failure. We also recommend switching the every 8-weeks dosing schedule of eculizumab with ravulizumab to minimize the exposure to COVID19 and other infections and reduce the economic burden on hospital systems due to less frequent dosing.

Given the worldwide COVID19 pandemic, specific clinical and economically cost-effective therapeutic options should be explored and prioritized. Treatment with ravulizumab during COVID19 pneumonia was safe, cost-effective, and found to have a favorable clinical course in a patient with PNH. A systemic prospective trial is warranted to demonstrate the utility and usefulness of ravulizumab in patients with PNH in the COVID-19 outbreak.

## Data Availability

Not applicable.
